# Treatment of Neuronopathic Mucopolysaccharidoses with Blood–Brain Barrier-Crossing Enzymes: Clinical Application of Receptor-Mediated Transcytosis

**DOI:** 10.3390/pharmaceutics14061240

**Published:** 2022-06-11

**Authors:** Hiroyuki Sonoda, Kenichi Takahashi, Kohtaro Minami, Toru Hirato, Tatsuyoshi Yamamoto, Sairei So, Kazunori Tanizawa, Mathias Schmidt, Yuji Sato

**Affiliations:** JCR Pharmaceuticals, Ashiya 659-0021, Hyogo, Japan; sonoda-h@jp.jcrpharm.com (H.S.); takahasi@jp.jcrpharm.com (K.T.); minami-k@jp.jcrpharm.com (K.M.); hirato-t@jp.jcrpharm.com (T.H.); t-yamamoto@jp.jcrpharm.com (T.Y.); sou-s@jp.jcrpharm.com (S.S.); tanizawa-k@jp.jcrpharm.com (K.T.); mschmidt@us.jcrpharm.com (M.S.)

**Keywords:** lysosomal storage disease, neuronopathic mucopolysaccharidosis, blood–brain barrier, neurodegeneration, enzyme replacement therapy, receptor-mediated transcytosis, transferrin receptor, insulin receptor

## Abstract

Enzyme replacement therapy (ERT) has paved the way for treating the somatic symptoms of lysosomal storage diseases (LSDs), but the inability of intravenously administered enzymes to cross the blood–brain barrier (BBB) has left the central nervous system (CNS)-related symptoms of LSDs largely impervious to the therapeutic benefits of ERT, although ERT via intrathecal and intracerebroventricular routes can be used for some neuronopathic LSDs (in particular, mucopolysaccharidoses). However, the considerable practical issues involved make these routes unsuitable for long-term treatment. Efforts have been made to modify enzymes (e.g., by fusing them with antibodies against innate receptors on the cerebrovascular endothelium) so that they can cross the BBB via receptor-mediated transcytosis (RMT) and address neuronopathy in the CNS. This review summarizes the various scientific and technological challenges of applying RMT to the development of safe and effective enzyme therapeutics for neuronopathic mucopolysaccharidoses; it then discusses the translational and methodological issues surrounding preclinical and clinical evaluation to establish RMT-applied ERT.

## 1. Introduction

Delivery of therapeutics to the brain has always been hampered by the blood–brain barrier (BBB), which protects the brain from external macromolecules, such as pathogenic and toxic substances [1]. Indeed, the brain has been touted as a ‘sanctuary’ against chemotherapy, because extravasating malignant cells escape from anti-cancer drugs in the peripheral blood stream, lurk in the brain, and eventually cause fatal metastases therein [2]. Various efforts have been made, therefore, to deliver therapeutics for brain diseases by circumventing the BBB, including temporary mechanical disruption of the BBB by hyperthermia [3] and ultrasound [4,5], and drug administration into the cerebrospinal fluid (CSF) by intrathecal [6,7] and intracerebroventricular (ICV) [8] routes, whereby the drugs are expected to diffuse through the CSF and become immersed in the brain parenchyma.

In contrast to the traditional view of the BBB as being made up primarily of restrictive endothelial tight junctions that sequester the systemic/peripheral blood flow and the brain, recent advances in BBB research have revealed that the BBB works more as a dynamic neurovascular unit that regulates the transport of substances [9]. Transcytosis is one such innate transport mechanism by the neurovascular unit, in particular, the vascular endothelial cells. Receptor-mediated transcytosis (RMT), as opposed to non-selective adsorptive transcytosis, has received attention as a promising pathway through which to traffic large molecules such as enzymes and biologics across the BBB [10].

Lysosomal storage diseases (LSDs) are a group of some 70 genetic metabolic disorders in which enzyme deficiencies in lysosomes cause systemic pathological accumulation of uncatabolyzed substrates, resulting in multisystemic progressive damage that manifests in a broad spectrum of debilitating symptoms, including coarse facies, hepatosplenomegaly, upper airway obstruction, cardiac dysfunction, and neurocognitive impairments [11], some of which are life-limiting. Successful development of recombinant enzyme therapeutics has enabled restoration of enzyme activities in some LSDs [12,13,14], and the advent of enzyme replacement therapy (ERT) has since led to significant improvements in somatic/peripheral symptoms and proximal outcomes in some LSDs [15]. However, as the BBB impedes the delivery of the therapeutic enzymes into the brain, conventional ERT cannot alleviate substance accumulation in the central nervous system (CNS), so progressive neurodegeneration in the CNS remains unbridled and culminates in neurocognitive deterioration [16]. Since most LSDs involve such unassailable CNS pathology, they are also known as neuronopathic LSDs, and to this day, the means to deliver enzyme therapeutics to the brain remains a critical unmet medical need [17,18].

For intra-CSF administration of therapeutic enzymes, the ICV route has proved viable, as exemplified by cerliponase alfa for the treatment of neuronal ceroid lipofuscinosis type 2 [19,20] and idursulfase beta for mucopolysaccharidosis II (MPS II, Hunter syndrome) [21]. However, even after successful drug distribution to the brain parenchyma following intra-CSF administration, significant drug efficacy is not yet fully guaranteed [22], because the CSF remains problematic as a vehicle for drug delivery [23]. Moreover, concomitant peripheral administration of enzyme therapeutics to deal with substance accumulations in the periphery is necessary, which imposes further burdens on pediatric patients and is inimical to lifelong treatment for these chronic ailments.

Consequently, an enzyme therapeutic designed to address both the peripheral/somatic and CNS manifestations will be the most suitable means of overcoming these difficulties and providing an improved form of ERT for neuronopathic LSDs. Several attempts have been made to realize these objectives by equipping enzyme therapeutics with the capacity to undergo RMT. This review summarizes the hitherto known applications of RMT for drug delivery to treat neuronopathic MPSs, in particular, illustrates the pitfalls and challenges of engineering enzyme therapeutics to harness effective RMT, highlights translational issues in establishing RMT-applied ERT by preclinical and clinical evaluations, and, finally, delineates the remaining issues surrounding drug delivery to the brain in general.

## 2. Transcytosis through the BBB: A Breakthrough for Brain Drug Delivery

### 2.1. Application of Receptor-Mediated Transcytosis for Brain Drug Delivery

Transcytosis is a mechanism of transcellular transport of molecules via vesicles [9,24] and contributes to the physiological function of the BBB in regulating substance transport from the systemic circulation to the CNS. When activated in cerebrovascular endothelial cells, this mechanism can also serve as a potential conduit for ferrying large molecules into the brain parenchyma. It has, therefore, kindled various research projects to apply it to brain drug delivery by what is known as the Trojan horse method [25], whereby a drug normally nontransferable across the BBB is, by chimeric peptide technology, conjugated or fused to a BBB transport vector, the vector being an endogenous peptide or anti-receptor monoclonal antibody that undergoes RMT without competing with endogenous ligands. RMT has been actively utilized in modifying enzyme therapeutics for ERT for neuronopathic LSDs [26,27]. This application has become possible thanks to the evolving understanding of intracellular trafficking, receptor binding, and protein engineering [28]. RMT is considered very suitable for application to pharmacotherapy [29] due to the innate physiological mechanism of the BBB that makes abundant receptors available, in addition to which, the very small inter-capillary distances in the brain allow each neuron to be perfused by the blood vessels surrounding it, making it ready to receive the trafficked substance [30]. Moreover, RMT allows highly specific trafficking of the targeted substance to which specific antibodies are fused. Therefore, RMT enables more stable and repeatable substance trafficking than modification of BBB permeability, which can be transient and perturb the normal function and structure of the BBB [29].

The main receptors studied for RMT so far are insulin [31,32,33] and transferrin receptors (TfRs) [34,35,36,37,38], although others (e.g., low-density lipoprotein receptor [39,40], neurotropic virulence factor receptors [41,42], CD98 heavy chain [43], and GLUT1 [44,45]) have also been suggested as potentially useful for brain drug delivery.

**Figure 1:**
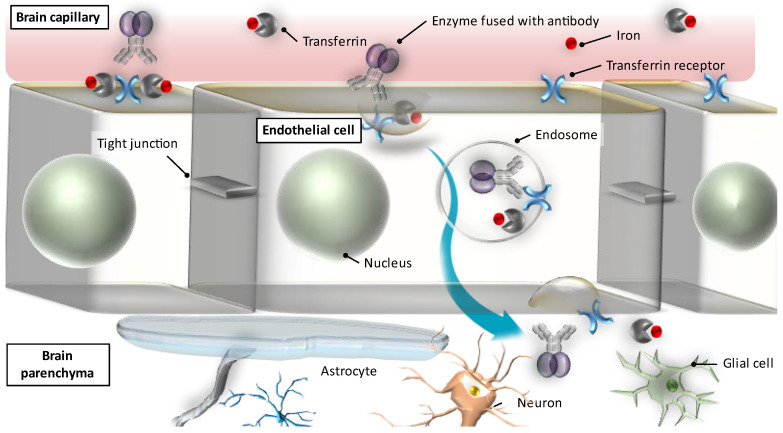
A schematic representation of transferrin receptor-mediated transcytosis in the blood–brain barrier (Revised from Yamamoto and Kawashima [46]).

Figure 1 illustrates the mechanism of transcytosis mediated by TfRs [46]. Transferrin binds to the TfRs located on the luminal side of the microvascular endothelial cells in the brain and is absorbed into the endothelial cells (endocytosis), in which it is then transported towards the abluminal side of the cells facing the brain, and subsequently released from the receptors to reach the brain parenchyma (exocytosis). Likewise, enzymes fused with anti- TfR antibodies bind to the TfRs, are then internalized into and trafficked across the endothelial cells, and are finally unleashed into the abluminal side of the endothelium so that they can reach the brain parenchyma to exert drug efficacy in the targeted sites of action (i.e., neurons and glial cells).

### 2.2. Optimizing RMT for ERT

Successful application of RMT for ERT can be realized, first, when attachment of the enzyme to the ‘Trojan horse’ (i.e., a molecular cargo on which to load the drug for delivery across the BBB) is achieved through high-standard genetic engineering [25], and, secondly, when critical factors affecting RMT [47] are modulated so as to enable the most efficient and stable trafficking of the drug. The former requires optimal molecular architecture of the enzyme therapeutic as a whole [48] with the help of precise protein and antibody engineering [24], whilst the latter requires elucidation of the mechanism of antibody passage across the BBB [29], which involves many hitherto unanswered questions.

#### 2.2.1. Antibody Engineering for RMT

In order to design and generate ideal antibodies for specific RMT, several serious limitations that have long compromised biotherapeutic engineering need to be overcome; these include poor pharmacokinetic parameters, non-optimal distribution, inhibition of their binding with Fc receptors, toxicity [49], and untoward influence on the original receptor functions. Furthermore, the immunogenicity inherent in biotherapeutics that is associated with risks of decreased tolerance and efficacy must be controlled if a therapeutic is to be utilized for long-term treatment, as is the case with ERT for neuronopathic MPS. Therefore, production of biotherapeutics requires continual optimization processes to maximize their therapeutic potential, on the one hand, and to ensure an acceptable safety profile on the other [50]. The optimization processes must also aim to achieve the most favorable pharmacodynamic response and pharmacokinetic parameters as possible. Furthermore, when a suitable antibody is tailored as a biotherapeutic for RMT-applied ERT targeting a specific neuronopathic MPS, the bioengineering processes may very well have to be revamped when a different enzyme needs to be fused with an antibody for separate ERT for a different LSD, even when the same previously established RMT mechanism is utilized. In other words, successful realization of RMT-applied ERT for a neuronopathic LSD depends heavily on whether specifically optimized design and production of an antibody-fused therapeutic can be achieved in the time and with the resources and bioengineering prowess available.

Goulatis [51] pointed out that antibody-antigen receptor interaction plays a pivotal role in optimizing substance trafficking across the BBB, which is deeply affected by binding affinity, avidity, and pH sensitivity. Ongoing controversies surround the optimal combination of antibody affinity towards the antigen receptor and the brain’s uptake of antibody-fused therapeutics. Whereas moderate-affinity monovalent anti-TfR antibodies fused with an enzyme therapeutic have been reported to traverse the BBB more efficiently than high-affinity bivalent antibodies fused with a comparable enzyme [48], antibodies with relatively high affinity have been shown to achieve efficient transport across a wide range of injection doses, as opposed to antibodies with low affinity transported only at high doses [52].

Most of the enzymes that are deficient in LSDs incorporate modified mannose-6-phosphatase (M6P) and undergo hepatic and other clearance dependent on M6P receptors (M6PRs), which accounts for their limited plasma retention time. This negatively affects their binding to the TfRs on cerebrovascular endothelial cells. In order to ensure sufficient binding to the TfRs, enzyme therapeutics need to have binding affinity higher than is generally seen in biologics with good plasma retention. However, when enzymes are released from endothelial cells into the brain parenchyma, high binding affinity invariably leads to reduced dissociation of the enzymes from the TfRs, thereby diminishing the number of molecules that reach the brain. Taken together, it seems sensible to strike a balance between the binding efficiency of enzyme therapeutics to the apical side of endothelial cells and their efficient dissociation on the vasolateral side at the same time to achieve the most suitable avidity for drug delivery across the BBB. Furthermore, accumulated clinical data from mid- to long-term ERT are needed to determine the optimal dosage and actual clinical efficacy of the therapeutics for neuronopathic LSDs, because such matters cannot be resolved by preclinical and theoretical discussions alone.

**Figure 2:**
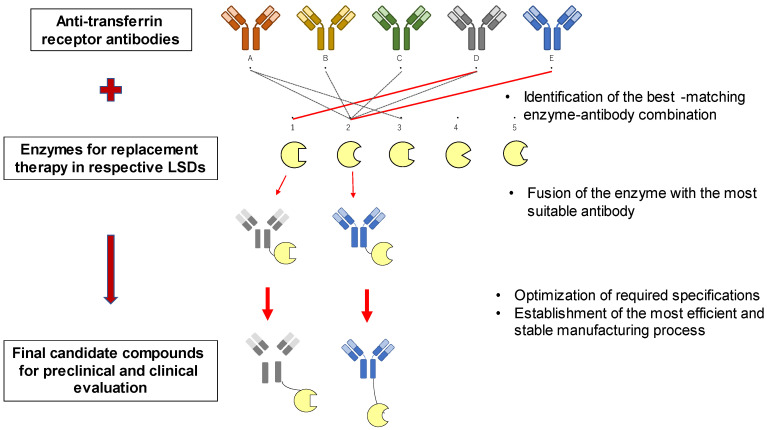
Schematic flow of optimization process of biotherapeutic for RMT-applied ERT.

After appropriate bioengineering methods for creating a biotherapeutic for RMT-applied ERT are duly established, sufficient quantities have to be manufactured for the drug to be tested in preclinical and clinical studies. This invariably involves timely scaling-up of drug production, with an eye to further augmentation to supply a wider patient population after the drug is approved. Unlike the preclinical and clinical studies of the therapeutic, the ingenious and painstaking efforts devoted to the bioengineering and manufacturing of the drug usually remain unpublished, because these endeavors involve essential information pertaining to intellectual property. Thus, discussion of these critical processes is inevitably restricted to mere general descriptions of the major issues without any quantitative or qualitative details. It would actually be very helpful if detailed descriptions of the caveats and other issues were published to avoid unnecessary repetition of mistakes, underestimation of critical points, and unguided guesswork in this unchartered field. Figure 2 summarizes the complex processes of selecting, optimizing, and manufacturing enzyme therapeutics for RMT-applied ERT for neuronopathic LSDs.

#### 2.2.2. Other Known Factors Regulating Transcytosis

Numerous factors are known to affect the transcytosis mechanism [47], with physiochemical factors playing an indispensable role, as seen in TfR-mediated transcytosis, which depends on the pH and polarity of proteins [53]. Temperature [54] and oxygen levels have also been suggested to influence transcytosis, the latter being observed, for instance, in BBB permeability affected by hypoxia [55]. Furthermore, several cytokines have been identified as related to transcytosis via cellular signaling amongst endothelial cells, pericytes, and astrocytes [56]. Muldoon et al. [57] postulated a physiological barrier at the basal lamina of the brain microvasculature distal to the anatomic BBB (tight junction), which limits the distribution of proteins and viral particles with large molecular weights after transvascular delivery to the brain. Although the roles of these factors in relation to transcytosis are far from being fully understood, their potential implications may require attention in optimizing RMT for ERT.

## 3. Preclinical Evaluation of RMT-Applied ERT

A PubMed literature search using enzyme replacement therapy and transcytosis as keywords produced only 10 hits for the past 10 years up to April 2022. Given this limited number of references, this section looks mainly at the preclinical and clinical studies of pabinafusp alfa (JR-141), a recently developed drug for RMT-applied ERT. Pabinafusp alfa is a genetically engineered fusion protein developed by JCR Pharmaceuticals for intravenous ERT for neuronopathic MPS II. It consists of an anti-human TfR (hTfR) antibody and human iduronate-2-sulfatase (IDS) fused to the C terminus of the immunoglobulin G (IgG) heavy chain. Robust preclinical [37,58,59,60,61] and clinical [62,63,64,65] evidence shows that it exhibits unequivocal dual efficacy against peripheral/somatic and CNS manifestations in patients with genetic IDS deficiency by delivering the enzyme therapeutic via TfR-mediated transcytosis across the BBB (a proprietary technology named J-Brain Cargo^®^). It received regulatory approval in Japan in 2021, spearheading other RMT-applied enzyme therapeutics in development worldwide.

### 3.1. Preclinical Efficacy Evaluation

Preclinical proof of concept of pabinafusp alfa in terms of its dual efficacy was examined stepwise [37]. First, human TfR-mediated cellular incorporation of the drug was shown in cultured human fibroblasts as in vitro evidence of its intracellular uptake via TfR-mediated endocytosis. Pabinafusp alfa was then administered intravenously to hTfR knock-in mice (an animal model of MPS II). While pabinafusp alfa was detected in the brain, naked hIDS was not, thereby providing in vivo evidence of delivery of the drug to the brain through the BBB. Finally, to underpin its dual efficacy, the enzyme activity of pabinafusp alfa was substantiated by observed reductions in the accumulation of substrates (i.e., glycosaminoglycans [GAGs]) in both the peripheral tissues and the brains of hTfR-knockin/Ids-knockout mice following intravenous administration of the drug.

Measurement of intracerebral GAG accumulations is the most direct indicator of enzyme activity in the brain, but because such measurement is inimical to clinical drug evaluation in patients, a surrogate efficacy endpoint had to be sought instead that could be used in both the preclinical and clinical investigations [58]. GAG concentrations (particularly those of heparan sulfate [HS]) in the CSF were found to correlate well with intracerebral GAG accumulations in the hTfR-knockin/Ids-knockout mice. An assay method to quantify HS concentrations by liquid chromatography-tandem mass spectrometry was established, which reliably measured HS accumulations in the CSF of the mice and demonstrated correlations between the intracerebral and intra-CSF HS levels. Furthermore, reductions in intracerebral and intra-CSF HS levels following intravenous administration of pabinafusp alfa, which had been observed previously in mouse brains, were replicated by this method in the hTfR-knockin/Ids-knockout mice, constituting preclinical proof of concept of the drug.

To further validate the efficacy of pabinafusp alfa against neurodegeneration, Morimoto et al. [59] demonstrated that the clearance of intracerebral HS accumulations induced by intravenous administration of pabinafusp alfa prevents neurodegeneration and resultant neurocognitive dysfunctions in MPS II mice. The drug reduced HS levels and attenuated histopathological changes in both the brain and peripheral tissues. Moreover, the loss of spatial learning abilities, a manifestation of neurocognitive impairment in MPS II mice, was completely suppressed by pabinafusp alfa, but not by idursulfase, indicating an association between HS deposition in the brain, neurodegeneration, and CNS manifestations. Furthermore, HS concentrations in the brain and their pabinafusp alfa-induced reduction correlated with those in the CSF. Dose-dependent relationships between long-term intravenous treatment with pabinafusp alfa and its effects on the CNS (reductions in HS levels in the brain and CSF, prevention of neuronal damage, and improved neurobehavioural performance) in the model mice [61] corroborates a quantitative dose-dependent relationship between HS reduction in the CNS and neurocognitive improvements in MPS II mice. Taken together, these preclinical findings establish the central efficacy of the drug in both stabilizing and preventing neuronopathy in MPS II.

Arguello et al. [48] have also reported positive results with an IDS transport vehicle (DNL 310) utilizing transferrin receptor-mediated transcytosis in MPS II mice: reduced levels of peripheral and CNS GAGs were noted along with improvements in auricular, skeletal, and neurobehavioural abnormalities, although the dose-response relationship between these effects and DNL 310 have apparently not yet been examined.

These preclinical evaluations have revealed a number of challenges that may be informative for similar future endeavors to establish RMT-applied ERT for neuronopathic LSDs at large. First, in contrast to the ostensibly straightforward pathophysiology common in LSDs (i.e., genetic enzyme deficiency causing accumulation of uncatabolized substrates that leads to systemic dysfunction), the exact neuropathogenesis is complex and remains to be elucidated [26]. Moreover, there is no established optimal method for evaluating novel therapeutics with new mechanisms of action against this complex and severe progressive disease. At the very least, the items listed below should be conceptualized, examined in practice, validated, and included in the evaluation process of novel therapeutics for neuronopathic LSDs. These items also need be shared and discussed with regulatory agencies to confirm their scientific, medical, and regulatory acceptability and ensure timely regulatory approval.

- Key pathognomonic signs and symptoms to be selected and focused on;
- Clinical efficacy endpoints that correlate well with these signs and symptoms and are considered most likely to respond to treatment;
- Surrogate endpoints that can represent and correlate with these clinical endpoints, and that are also measurable in animal models of the disease;
- Quantitative and qualitative methods to evaluate these endpoints: these methods also need to be conducive to both preclinical and clinical studies.

### 3.2. Preclinical Safety Evaluation

Antibody-based drugs generally have high target specificity [66], as seen in the complement-dependent cytotoxicity (CDC) and antibody-dependent cellular cytotoxicity (ADCC) of biotherapeutics for cancer [67,68]. However, in therapeutic areas outside oncology, cytotoxicity mediated by the antibody would merely compromise the safety of the biotherapeutic and be counterproductive to its expected efficacy. On the other hand, whether the physiological functions of the innate receptors specifically utilized in RMT can be affected by their utilization needs to be evaluated, particularly when the drug is used long-term. Iron and glucose metabolism, for instance, should be examined for its potential untoward influence on ERT via the transferrin and insulin receptors, respectively.

#### 3.2.1. Evaluation of Antibody-Derived Cytotoxicity

A four-week repeat-dose toxicity study of pabinafusp alfa at dosages of up to 30 mg/kg/week was conducted in sexually mature and juvenile monkeys, along with a 26-week repeat-dose study in 2- to 4-year-old monkeys [60]. CDC (an effector function of IgG and IgM) is a cytolytic cascade triggered by binding of the complement C1q to the constant region of cell-bound antibodies, followed by activation of a series of complement proteins [69,70]. CDC was elicited by the anti-hTfR monoclonal antibodies (mAb) used in pabinafusp alfa, but not by the fusion protein as a whole. This is probably because the fusion of the enzyme to the mAb interferes with the access of complement proteins to the antibody moiety via steric hindrance, hence the loss of CDC activity of pabinafusp alfa. ADCC (another effector function of IgG) is involved in the cytotoxicity towards opsonized cells (cells coated with antibodies) of such effector cells in the immune system as natural killer cells [ibid]. Thus, ADCC requires an interaction between the antibody CDR domains and antigens on the target cells (i.e., endothelial cells) along with binding of the antibody Fc domains to the Fcγ receptors on the effector cells. Neither the humanized anti-hTfR mAb nor pabinafusp alfa caused ADCC in this toxicology study [60]. The carbohydrate structure of the Fc region is known to be critical for the binding of antibodies to the Fcγ receptors of the effector cells [ibid]. As the structure of the anti-TfR mAb moiety in pabinafusp alfa is similar to that of natural IgG1, the binding ability of pabinafusp alfa to the Fcγ receptors may be retained at least to some degree. Therefore, the bridge formation between the effector cells and the endothelial cells, an essential step in initiating ADCC, is lacking, thus precluding safety concerns regarding ADCC. 

#### 3.2.2. Potential Influence of RMT on the Original Receptor Functions

The receptor-binding property of an antibody-fused therapeutic for RMT-applied ERT may raise concerns about its influence through the antibody-receptor interaction on the original physiological functions of the receptors. Indeed, a clinical trial of AGT-181 (valanafusp alpha), α-L-iduronidase fused with anti-insulin receptor antibody, found drug-related transient hypoglycemia in 6.4% of the patients with MPS I [71]. However, this finding can be attributed to the insulin agonist activity of the anti-insulin receptor antibody that constitutes AGT-181 [72], and is not necessarily related to the transcytotic effect per se of the compound. In a clinical trial of DNL-310, an enzyme fusion protein that contains a low-affinity transferrin-binding peptide, anemia was detected in two of the five patients with MPS II given DNL-310, although this was not considered to be related to the drug [73]. However, the potential association of DNL-310 with anemia in relation to the transferrin receptors, also expressed in high amounts on erythroblasts, may require further evaluation.

In contrast, preclinical studies of pabinafusp alfa show no interference with the binding of transferrin to its receptors [60]. We consider this to be because the TfR epitope recognized by the anti-TfR antibody in pabinafusp alfa is distinct from the transferrin binding site of the TfR. More importantly, cynomolgus monkeys treated with pabinafusp alfa showed no abnormalities in such iron-related parameters as serum iron, unsaturated iron binding capacity, ferritin, haptoglobin, and total iron binding capacity [ibid]. Thus, pabinafusp alfa has minimal potential to produce any toxicity related to perturbation of the iron metabolism. The clinical trials of pabinafusp alfa so far conducted, as detailed below, have elicited no adverse events associated with the iron metabolism [62,63,64,65].

Overall, the likelihood of potential interactions between the antibody in the RMT-applied therapeutic and the targeted receptor seems to differ from one therapeutic to another, and is, perhaps, not dependent on the kind of receptor that mediates transcytosis. Furthermore, preclinically identified interactions may not directly translate into relevant or clinically significant adverse events, while preclinically undetected interactions may very well lead to receptor-associated adverse events. Translating preclinical toxicological findings into clinically meaningful safety endpoints is never easy, but given the debilitating nature of progressive neurodegeneration in neuronopathic LSDs, for which no ERT is available, the potential risks of these events are almost certainly outweighed by the clinical benefits of RMT-applied ERT, provided that adverse events related to RMT, if any, are clinically manageable and do not offset the overall benefits of the treatment.

#### 3.2.3. Anti-Drug Antibodies and Resultant Infusion-Associated Reactions

As detailed in Section 4.3.3, repeated enzyme replacement is known to generate anti-drug antibodies that can lead to infusion-associated reactions (IARs) that are detrimental to safety and efficacy. Furthermore, cross-reactive immunological material (CRIM)-negative LSD patients with complete absence of enzyme activity are known to frequently exhibit IARs caused by neutralizing antibodies in particular. While the anti-drug antibodies and enzyme therapeutics can be trafficked together to intracellular lysosomes in which the enzyme can function once the antibody degrades, the neutralizing antibodies inhibit the M6PR-dependent intracellular uptake of the enzyme, significantly reducing drug efficacy. However, this is not the case with fusion proteins such as pabinafusp alfa, which is expected to allow TfR-dependent cellular uptake, as long as no neutralizing antibodies against the antibody CDR domain are generated, even if anti-drug antibodies against the enzyme or neutralizing antibodies against M6PRs are present. Such a mechanism of action involving both TfRs and M6PRs is, therefore, expected to contribute to reducing the risk of neutralizing antibody generation and ensuring better safety and efficacy in long-term ERT.

## 4. Translation and Clinical Establishment of RMT-Applied ERT

### 4.1. RMT-Applied ERT

Table 1 summarizes the clinical development of five therapeutics to which RMT has been applied in order to establish ERT for neuronopathic MPS. AGT-181 and AGT-182 utilize the insulin receptor, while the other three harness RMT via the TfR.

### 4.2. Issues with Extrapolating Preclinical Findings to Humans

The antibodies utilized for enzyme therapeutics in RMT-applied ERT are designed to bind to the innate human receptors on the cerebrovascular endothelial cells. However, preclinical efficacy and safety evaluations of the therapeutics have to be conducted in animal models (mice, rats, and monkeys). Although inter-species differences in the amino acid sequence of the TfR may be relatively small, subtle differences in the epitope of the TfR can have a significant influence on the antibody affinity of the biotherapeutic. Therefore, hTfR-knockin/Ids-knockout mice were required, as they enable comparable and extrapolatable efficacy evaluation. However, as genetic induction in rats and monkeys is prohibitively difficult, the extrapolability of the preclinical data on these animals depends on the extent to which the antibody affinity of the biotherapeutics to their respective TfRs is reasonably comparable to that in humans. This general issue of scientific appropriateness and regulatory acceptability of extrapolated preclinical data is of particular importance for RMT-applied ERT in terms of pharmacokinetics and pharmacodynamics, as detailed below.

#### 4.2.1. Pharmacodynamic Issues

Higher brain functions (e.g., memory, consciousness, emotion, and cognition) and their disorders are notoriously difficult to evaluate in animal experiments and, even if it is possible, further difficulties arise in extrapolating the preclinical data to humans, hence the low clinical success rate for drugs that show tremendous promise in animal experiments intended to model psychiatric pathophysiology [74,75]. As it is all but impossible to comprehensively capture the multifaceted manifestations of neuronopathic LSDs either preclinically or clinically [26], some key features must be selected, taking account of their clinical significance, quantifiability, correlation with treatment response, operational feasibility, and regulatory acceptability. For this purpose, a two-pronged approach to pharmacodynamic evaluation has been taken: (1) measuring GAG levels (in particular HS levels) in the CSF as a representative etiological factor for the subsequent neuropathological cascade of events, and (2) conducting evaluation and analysis of neurocognitive development as one of the most notable clinical correlates in the final stage of the cascade. The latter was done preclinically by using the Morris water maze test for spatial learning assessment [59], and clinically by employing reliable and validated neurodevelopmental batteries for neurocognitive assessment [63,64,65], in addition to other quantitative and qualitative measures, such as histopathological assessment of the brain for preclinical evaluation, and magnetic resonance imaging of the brain in MPS II patients. These pharmacodynamic evaluations may be applicable to other types of neuronopathic MPS, but they probably need to be modified or supplanted with other disease-specific assessments for individual neuronopathic LSDs, given the marked heterogeneity in their manifestations, clinical courses, outcomes, and severity [11].

In our preclinical studies with mouse disease models, we have often observed markedly aggressive behavior (e.g., fighting) in caged animals (unpublished). Such behavioral findings may be of translational importance, because they may correspond to neurocognitive impairments and associated behavioral features that have been well documented in MPS II patients. Indeed, throughout our clinical trials in Japan and Brazil, many patients exhibited anxiety, excitability, and hyperactivity that often rendered even sitting at a table for meals difficult and family outings impossible. As the trials continued, narrative reports from physicians, caregivers, and family members described markedly improved mood, emotional stability, comprehension, and responsiveness in the patients, some of whom then even managed to stay calm during mealtimes in and outside their homes [62,63,64,65,76]. These positive behavioral changes often seem to be preceded by subtle yet important non-verbal aspects such as smiling, which, as an attachment behavior, is regarded as a critical developmental milestone in child development [77]. These behavioral changes may be taken as early favorable signs that foreshadow later, more recognizable CNS function-related responses to treatment. In turn, the aforementioned specific behavioral characteristics observed in the mouse models may be worth further attention, as they might potentially serve as preclinical endpoints for capturing early treatment response.

#### 4.2.2. Pharmacokinetic Issues

In intrathecal and intracerebroventricular administration of enzyme therapeutics for delivery to the brain, the CSF in the subarachnoid space and the ventricles assumes an essential pharmacokinetic role as a medium for drug administration, distribution, and elimination. In allowing the substrate concentrations therein to serve as a critical surrogate efficacy endpoint, as described above, the CSF is also of pharmacodynamic importance. However, RMT-applied ERT differs from ERT via intra-CSF drug administration in terms of the role of the CSF. In the former, the drug is distributed through the systemic blood circulation; RMT then occurs, followed by drug diffusion towards the brain parenchyma. In this process, the CSF works more or less as a reservoir into which a very limited portion of the drug is excreted after being consumed, making the intra-CSF drug concentrations negligibly small. In contrast, drug concentrations in the CSF are naturally very high after intra-CSF drug administration, although smaller quantities of the drugs actually reach the brain parenchyma than in RMT-applied ERT. This is because drugs administered via the CSF must be distributed in retrograde (i.e., cephalad or headward) diffusion against the normal CSF flow to reach the brain parenchyma, so drug penetration from the CSF can be minimal [1,78]. Thus, interpretation of the CSF-related pharmacokinetic parameters must take account of the unique characteristics of the CSF circulation in relation to drug delivery. Here again, the extrapolability of the CSF findings and related pharmacokinetic parameters in experimental animals needs further attention [79].

### 4.3. Clinical Investigation with RMT-Applied ERT for Neuronopathic LSDs

#### 4.3.1. Overall Challenges

The general challenges for clinical trials involving rare diseases include paucity of patients available for trials, limited enrollment capacity/efficiency at investigational sites, limited numbers of investigators/specialists globally, and operational hurdles to conducting multinational multi-site trials. It follows that the oft-employed strategy of increasing the number of participating sites to compensate for the limited number of patients at each one is not as helpful when it comes to clinical trials involving rare diseases as it is in other therapeutic areas. Neuronopathic LSDs present further problems related mostly to their complex and progressive pathology, which renders efficacy evaluation and subsequent determination of therapeutic effects difficult and time-consuming. One counterintuitive challenge is that even though neuronopathic LSDs are rare, they exhibit enormous phenotypic heterogeneity, which partly accounts for their frequently delayed diagnosis and treatment initiation. These factors only further complicate the constraints involved in designing and conducting clinical trials, making the gold standard of clinical trials—a sufficiently powered comparative randomized controlled trial—enormously challenging to conduct in a timely and operationally realistic fashion. Furthermore, when there is no established standard treatment at all for the disease, an untreated comparator arm is not ethically acceptable. In such cases, one sensible option for making informative efficacy comparisons is to compare the treatment group with what is known as a historical control, which means using clinical data on previous patients with the same disease. It is necessary to ensure, of course, that both datasets are collected using the same, or at least comparable, assessment methods to allow meaningful efficacy analysis [80].

#### 4.3.2. Clinical Efficacy Evaluation

Treatment for neuronopathic LSDs must address both the peripheral/somatic and CNS manifestations, so both of these efficacy endpoints need to be captured quantitatively and qualitatively.

Peripheral efficacy endpoints focus on major somatic signs and symptoms (urinary and plasma concentrations of substrates, hepatosplenomegaly, cardiac and pulmonary functions, 6-minute walk test, and joint motion), while central efficacy evaluation needs to examine some major aspects of the neuronopathy that affect the manifold functions and structures of the CNS. The trials described above (completed or ongoing) have commonly examined substrate levels in the CSF, along with various neuropsychological functions via such established test batteries as the Bayley Scales of Infant and Toddler Development, the Kaufman Assessment Battery for Children, and the Vineland Adaptive Behavior Scales [63,64,65]. Neuroimaging is also necessary to identify cerebral atrophy, ventricular enlargement, and other discernible structural changes of the brain, and to assess visual and auditory functions.

One fundamental difficulty inherent in neurodevelopmental assessment is that while normal development always takes years, in patients with neuronopathic LSDs, it can proceed without marked disturbance from birth, reach a plateau in early childhood, and then deteriorate afterwards, albeit with marked variability [81]. Therefore, evaluations of the potential effects of RMT-applied ERT on developmental trajectories in patients with notable interindividual differences will invariably require observations over several years. This seriously compromises the mission to develop novel therapeutics expeditiously for patients with deteriorating neuronopathy, although it should also be noted that with short-term observation there is a risk of overlooking treatment efficacy that might have been noted with sufficiently long observation. A sensible compromise between the scientific requirement to establish long-term efficacy and the critical clinical need to expedite the introduction of novel treatments would be to use neurodevelopmental batteries for several years to evaluate neurocognitive efficacy along with surrogate endpoints to buttress positive CNS efficacy signals, and then to seek conditional regulatory approval with the data obtained, which should later be corroborated by long-term developmental and other CNS-related data collected in post-marketing surveys.

#### 4.3.3. Clinical Safety Evaluation

For RMT-applied ERT, two groups of safety endpoints require particular attention: adverse events related to the infusion of biotherapeutics (i.e., IARs) [82], and adverse events related to the effects of the test drugs on the specific innate receptors utilized for RMT. IARs are a set of common adverse drug reactions to monoclonal antibodies, and they involve various symptoms ranging from discomfort, skin and/or mucosal tissue manifestations (e.g., generalized hives, pruritis, and flushing), and gastrointestinal symptoms (nausea and vomiting), to more severe symptoms, such as respiratory compromise (dyspnea, wheezing, and hypoxemia) and hypotension. Serious manifestations, such as anaphylaxis and cytokine release syndrome, are sometimes reported, and these can be fatal if not properly managed. IRAs generally occur on the first day of drug administration, often starting within seconds or minutes of the first exposure. Premedication with antihistamines or corticosteroids, for example, is sometimes given to prevent or ease their occurrence, and slowing the infusion rate can also be effective. However, immunogenicity is a concern in enzyme replacement therapy [83], and various measures have been proposed to mitigate the long-term risk of immunogenicity and to ensure the therapeutic benefit of ERT for patients with LSDs (e.g., prophylactic immune tolerance induction). Such measures are necessary, because IARs affect not only the long-term tolerability and safety profile of the drug, but also the efficacy of the drug itself, which is why concurrent immunomodulation is sometimes recommended to maximize the efficacy of ERT [84].

The potential adverse events related to the specific receptors utilized for RMT-applied ERT are discussed in Section 3.2.2.

#### 4.3.4. Post-Approval Evaluations

Regulatory approval of RMT-applied ERT for neuronopathic LSDs on the basis of relatively limited efficacy data is necessary to meet the urgent medical needs of affected patients, but this means that long-term efficacy data are of particular importance to consolidate the effects of the drug on the CNS and ensure that the regulatory conditions for its approval are fully met. Furthermore, because neuronopathic LSDs are rare yet heterogenous as a nosological entity, much about them remains to be elucidated. It is very likely, therefore, that clinical data and observations from both pre- and post-approval studies will shed new light on the pathogenesis, clinical course, and prognosis of the disease by, for instance, differentiating patients with the same diagnosis according to their treatment response, thereby revealing hitherto unknown characteristics of the disease. Information thus obtained will facilitate accurate and timely diagnosis of the disease, which will, in turn, enable early initiation of the most appropriate treatment. The intertwined yet reciprocal relationship between diagnosis and treatment seen in LSDs, whereby a novel treatment helps refine diagnosis and vice versa, is reminiscent of the ancient clinical approach known as *diagnosis ex juvantibus* (attempting to diagnose a disease by treating it) [85].

## 5. Conclusions

This review summarizes some of the trailblazing efforts made to apply RMT to ERT for the treatment of neuronopathic MPSs. After more than 25 years and umpteen attempts to develop biotherapeutics for brain diseases, the possibility of using transcytotic mechanisms to reengineer biologics has been greeted with great excitement [86]. However, as the targeted disease itself and the application of RMT both involve innumerable unclarified issues, these efforts have confronted formidable obstacles. To overcome these, a relentless process of trial and error leading to ingenious ideas for improvement and serendipitous discoveries was required, and the true innovative originality of many of these advances remains unacknowledged. Indeed, some of the preclinical and clinical findings gained in establishing RMT-applied ERT [1] may, when put in a wider context, clear up many of the historical misconceptions about the CSF, the BBB, and the delivery of drugs to the brain [30].

Nevertheless, the efficacy of RMT-applied ERT against CNS symptoms requires long-term evaluation. Furthermore, in addition to its inability to treat neuronopathy, conventional ERT suffers from other notable limitations, such as lack of effect on the cardiac valves, trachea and bronchi, ears, and eyes, due to its limited ability to penetrate these tissues [87]. Notably, respiratory failure, which is the most common cause of death in patients with MPS II [88], is associated with obstructions caused by GAG deposits in the respiratory tract, which neither conventional ERT nor RMT-applied ERT can address sufficiently. Therefore, further research and improvement of ERT is called for. Firstly, it needs to be fortified so that the substance accumulations outside the reach of current ERT can be addressed in order to improve survival outcomes. Secondly, the effects of RMT-applied ERT on neuronopathy in MPS I and II need to be corroborated and established, beyond which it must then be applied to other neuronopathic LSDs. Last, but not least, complex development and provision of RMT-applied ERT can lead to considerable socioeconomic burden on healthcare at large. Hopefully, RMT will enable brain delivery of a wider range of therapeutics for other neurodegenerative diseases, many of which still defy treatment, in a sustainable and approachable manner to the patients and their caregivers.

## Figures and Tables

**Table 1:** Clinical trials of the new therapeutics for neuronopathic MPS that utilize receptor-mediated transcytosis.

Disease	Compound	Clinical Phase/Status	Targeted Receptors	Sponsor	Publication	Identifier
MPS I	AGT-181 (valanafusp alpha)	Phase I (completed)	Insulin receptors	ArmaGen	Giugliani et al. [70]	NCT02262338
	JR-171 (lepunafusp alfa)	Phase I (completed)	Transferrin receptors	JCR Pharmaceuticals	Not available	NCT04227600
MPS II	AGT-182	Phase I/II (completed)	Insulin receptors	ArmaGen	Not available	NCT03053089
	Pabinafusp alfa (JR-141)	Phase III (completed) Approved in Japan in 2021	Transferrin receptors	JCR Pharmaceuticals	Okuyama et al. [62,63] Giugliani et al. [65]	NCT03568175
		Phase II/III (completed) Filed for regulatory approval in Brazil in 2021			Giugliani et al. [64]	NCT03359213
		Phase III (recruiting in the US, EU, UK and Brazil)			Not available	NCT04573023
	DNL-310	Phase I/II (recruiting)	Transferrin receptors	Denali Therapeutics	Not available	NCT04251026

## Data Availability

Not applicable.

## References

[1] Sato Y., Minami K., Hirato T., Tanizawa K., Sonoda H., Schmidt M. (2022). Drug delivery for neuronopathic lysosomal storage diseases: Evolving roles of the blood brain barrier and cerebrospinal fluid. Metab. Brain Dis..

[2] Wilhelm I., Fazakas C., Molnar K., Vegh A.G., Hasko J., Krizbai I.A. (2018). Foe or friend? Janus-faces of the neurovascular unit in the formation of brain metastases. J. Cereb. Blood Flow Metab..

[3] Leuthardt E.C., Duan C., Kim M.J., Campian J.L., Kim A.H., Miller-Thomas M.M., Shimony J.S., Tran D.D. (2016). Hyperthermic Laser Ablation of Recurrent Glioblastoma Leads to Temporary Disruption of the Peritumoral Blood Brain Barrier. PLoS ONE.

[4] Lipsman N., Meng Y., Bethune A.J., Huang Y., Lam B., Masellis M., Herrmann N., Heyn C., Aubert I., Boutet A. (2018). Blood-brain barrier opening in Alzheimer’s disease using MR-guided focused ultrasound. Nat. Commun..

[5] Abrahao A., Meng Y., Llinas M., Huang Y., Hamani C., Mainprize T., Aubert I., Heyn C., Black S.E., Hynynen K. (2019). First-in-human trial of blood-brain barrier opening in amyotrophic lateral sclerosis using MR-guided focused ultrasound. Nat. Commun..

[6] Muenzer J., Hendriksz C.J., Fan Z., Vijayaraghavan S., Perry V., Santra S., Solanki G.A., Mascelli M.A., Pan L., Wang N. (2016). A phase I/II study of intrathecal idursulfase-IT in children with severe mucopolysaccharidosis II. Genet. Med..

[7] Jones S.A., Breen C., Heap F., Rust S., de Ruijter J., Tump E., Marchal J.P., Pan L., Qiu Y., Chung J.K. (2016). A phase 1/2 study of intrathecal heparan-N-sulfatase in patients with mucopolysaccharidosis IIIA. Mol. Genet. Metab..

[8] Matsuoka K., Tamura T., Tsuji D., Dohzono Y., Kitakaze K., Ohno K., Saito S., Sakuraba H., Itoh K. (2011). Therapeutic potential of intracerebroventricular replacement of modified human beta-hexosaminidase B for GM2 gangliosidosis. Mol. Ther..

[9] Ayloo S., Gu C. (2019). Transcytosis at the blood-brain barrier. Curr. Opin. Neurobiol..

[10] Banks W.A. (2016). From blood-brain barrier to blood-brain interface: New opportunities for CNS drug delivery. Nat. Rev. Drug Discov..

[11] Mehta A., Winchester B. (2012). Lysosomal Storage Disorders: A Practical Guide.

[12] Platt F.M. (2018). Emptying the stores: Lysosomal diseases and therapeutic strategies. Nat. Rev. Drug Discov..

[13] Kelly J.M., Bradbury A., Martin D.R., Byrne M.E. (2017). Emerging therapies for neuropathic lysosomal storage disorders. Prog. Neurobiol..

[14] Solomon M., Muro S. (2017). Lysosomal enzyme replacement therapies: Historical development, clinical outcomes, and future perspectives. Adv. Drug Deliv. Rev..

[15] Beck M. (2018). Treatment strategies for lysosomal storage disorders. Dev. Med. Child Neurol..

[16] Giugliani R., Vairo F., Kubaski F., Poswar F., Riegel M., Baldo G., Saute J.A. (2018). Neurological manifestations of lysosomal disorders and emerging therapies targeting the CNS. Lancet Child Adolesc. Health.

[17] Begley D., Scarpa M., Mehta A., Winchester B. (2012). Central Nervous System Aspects, Neurodegeneration and the Blood-Brain Barrier. Lysosomal Storage Disorders: A Practical Guide.

[18] Scarpa M., Orchard P.J., Schulz A., Dickson P.I., Haskins M.E., Escolar M.L., Giugliani R. (2017). Treatment of brain disease in the mucopolysaccharidoses. Mol. Genet. Metab..

[19] Schulz A., Ajayi T., Specchio N., de Los Reyes E., Gissen P., Ballon D., Dyke J.P., Cahan H., Slasor P., Jacoby D. (2018). Study of Intraventricular Cerliponase Alfa for CLN2 Disease. N. Engl. J. Med..

[20] De Los Reyes E., Lehwald L., Augustine E.F., Berry-Kravis E., Butler K., Cormier N., Demarest S., Lu S., Madden J., Olaya J. (2020). Intracerebroventricular Cerliponase Alfa for Neuronal Ceroid Lipofuscinosis Type 2 Disease: Clinical Practice Considerations From US Clinics. Pediatr. Neurol..

[21] Seo J.H., Kosuga M., Hamazaki T., Shintaku H., Okuyama T. (2021). Impact of intracerebroventricular enzyme replacement therapy in patients with neuronopathic mucopolysaccharidosis type II. Mol. Ther. Methods Clin. Dev..

[22] Naseri Kouzehgarani G., Feldsien T., Engelhard H.H., Mirakhur K.K., Phipps C., Nimmrich V., Clausznitzer D., Lefebvre D.R. (2021). Harnessing cerebrospinal fluid circulation for drug delivery to brain tissues. Adv. Drug Deliv. Rev..

[23] Abbott N.J., Pizzo M.E., Preston J.E., Janigro D., Thorne R.G. (2018). The role of brain barriers in fluid movement in the CNS: Is there a ‘glymphatic’ system?. Acta Neuropathol..

[24] Abdul Razzak R., Florence G.J., Gunn-Moore F.J. (2019). Approaches to CNS Drug Delivery with a Focus on Transporter-Mediated Transcytosis. Int. J. Mol. Sci..

[25] Pardridge W.M. (2002). Drug and gene targeting to the brain with molecular Trojan horses. Nat. Rev. Drug Discov..

[26] Sato Y., Okuyama T. (2020). Novel Enzyme Replacement Therapies for Neuropathic Mucopolysaccharidoses. Int. J. Mol. Sci..

[27] Terstappen G.C., Meyer A.H., Bell R.D., Zhang W. (2021). Strategies for delivering therapeutics across the blood-brain barrier. Nat. Rev. Drug Discov..

[28] Pulgar V.M. (2018). Transcytosis to Cross the Blood Brain Barrier, New Advancements and Challenges. Front. Neurosci..

[29] Kouhi A., Pachipulusu V., Kapenstein T., Hu P., Epstein A.L., Khawli L.A. (2021). Brain Disposition of Antibody-Based Therapeutics: Dogma, Approaches and Perspectives. Int. J. Mol. Sci..

[30] Pardridge W.M. (2016). CSF, blood-brain barrier, and brain drug delivery. Expert Opin. Drug Deliv..

[31] Boado R.J., Zhang Y., Zhang Y., Pardridge W.M. (2007). Humanization of anti-human insulin receptor antibody for drug targeting across the human blood-brain barrier. Biotechnol. Bioeng..

[32] Boado R.J., Hui E.K., Lu J.Z., Pardridge W.M. (2010). Drug targeting of erythropoietin across the primate blood-brain barrier with an IgG molecular Trojan horse. J. Pharmacol. Exp. Ther..

[33] Boado R.J., Ka-Wai Hui E., Zhiqiang Lu J., Pardridge W.M. (2014). Insulin receptor antibody-iduronate 2-sulfatase fusion protein: Pharmacokinetics, anti-drug antibody, and safety pharmacology in Rhesus monkeys. Biotechnol. Bioeng..

[34] Friden P.M., Walus L.R., Musso G.F., Taylor M.A., Malfroy B., Starzyk R.M. (1991). Anti-transferrin receptor antibody and antibody-drug conjugates cross the blood-brain barrier. Proc. Natl. Acad. Sci. USA.

[35] Fu A., Hui E.K., Lu J.Z., Boado R.J., Pardridge W.M. (2011). Neuroprotection in stroke in the mouse with intravenous erythropoietin-Trojan horse fusion protein. Brain Res..

[36] Bien-Ly N., Yu Y.J., Bumbaca D., Elstrott J., Boswell C.A., Zhang Y., Luk W., Lu Y., Dennis M.S., Weimer R.M. (2014). Transferrin receptor (TfR) trafficking determines brain uptake of TfR antibody affinity variants. J. Exp. Med..

[37] Sonoda H., Morimoto H., Yoden E., Koshimura Y., Kinoshita M., Golovina G., Takagi H., Yamamoto R., Minami K., Mizoguchi A. (2018). A Blood-Brain-Barrier-Penetrating Anti-human Transferrin Receptor Antibody Fusion Protein for Neuronopathic Mucopolysaccharidosis II. Mol. Ther..

[38] Ullman J.C., Arguello A., Getz J.A., Bhalla A., Mahon C.S., Wang J., Giese T., Bedard C., Kim D.J., Blumenfeld J.R. (2020). Brain delivery and activity of a lysosomal enzyme using a blood-brain barrier transport vehicle in mice. Sci. Transl. Med..

[39] May P., Woldt E., Matz R.L., Boucher P. (2007). The LDL receptor-related protein (LRP) family: An old family of proteins with new physiological functions. Ann. Med..

[40] Demeule M., Currie J.C., Bertrand Y., Che C., Nguyen T., Regina A., Gabathuler R., Castaigne J.P., Beliveau R. (2008). Involvement of the low-density lipoprotein receptor-related protein in the transcytosis of the brain delivery vector angiopep-2. J. Neurochem..

[41] Kumar P., Wu H., McBride J.L., Jung K.E., Kim M.H., Davidson B.L., Lee S.K., Shankar P., Manjunath N. (2007). Transvascular delivery of small interfering RNA to the central nervous system. Nature.

[42] Huey R., Hawthorne S., McCarron P. (2017). The potential use of rabies virus glycoprotein-derived peptides to facilitate drug delivery into the central nervous system: A mini review. J. Drug Target..

[43] Zuchero Y.J., Chen X., Bien-Ly N., Bumbaca D., Tong R.K., Gao X., Zhang S., Hoyte K., Luk W., Huntley M.A. (2016). Discovery of Novel Blood-Brain Barrier Targets to Enhance Brain Uptake of Therapeutic Antibodies. Neuron.

[44] Anraku Y., Kuwahara H., Fukusato Y., Mizoguchi A., Ishii T., Nitta K., Matsumoto Y., Toh K., Miyata K., Uchida S. (2017). Glycaemic control boosts glucosylated nanocarrier crossing the BBB into the brain. Nat. Commun..

[45] Xie J., Gonzalez-Carter D., Tockary T.A., Nakamura N., Xue Y., Nakakido M., Akiba H., Dirisala A., Liu X., Toh K. (2020). Dual-Sensitive Nanomicelles Enhancing Systemic Delivery of Therapeutically Active Antibodies Specifically into the Brain. ACS Nano.

[46] Yamamoto R., Kawashima S. (2022). [Pharmacological property, mechanism of action and clinical study results of Pabinafusp Alfa (Genetical Recombination) (IZCARGO((R)) I.V. Infusion 10 mg) as the therapeutic for Mucopolysaccharidosis type-II (Hunter syndrome)]. Nihon Yakurigaku Zasshi.

[47] Tjakra M., Wang Y., Vania V., Hou Z., Durkan C., Wang N., Wang G. (2019). Overview of Crosstalk Between Multiple Factor of Transcytosis in Blood Brain Barrier. Front. Neurosci..

[48] Arguello A., Mahon C.S., Calvert M.E.K., Chan D., Dugas J.C., Pizzo M.E., Thomsen E.R., Chau R., Damo L.A., Duque J. (2022). Molecular architecture determines brain delivery of a transferrin receptor-targeted lysosomal enzyme. J. Exp. Med..

[49] Zvonova E., Tyurin A., Soloviev A. (2018). Strategies for Modulation of Pharmacokinetics of Recombinant Therapeutic Proteins. Biol. Bull. Rev..

[50] Kuramochi T., Igawa T., Tsunoda H., Hattori K. (2014). Humanization and simultaneous optimization of monoclonal antibody. Methods Mol. Biol..

[51] Goulatis L.I., Shusta E.V. (2017). Protein engineering approaches for regulating blood-brain barrier transcytosis. Curr. Opin. Struct. Biol..

[52] Pardridge W.M., Chou T. (2021). Mathematical Models of Blood-Brain Barrier Transport of Monoclonal Antibodies Targeting the Transferrin Receptor and the Insulin Receptor. Pharmaceuticals.

[53] Sade H., Baumgartner C., Hugenmatter A., Moessner E., Freskgard P.O., Niewoehner J. (2014). A human blood-brain barrier transcytosis assay reveals antibody transcytosis influenced by pH-dependent receptor binding. PLoS ONE.

[54] Delvendahl I., Vyleta N.P., von Gersdorff H., Hallermann S. (2016). Fast, Temperature-Sensitive and Clathrin-Independent Endocytosis at Central Synapses. Neuron.

[55] Bailey D.M., Bain A.R., Hoiland R.L., Barak O.F., Drvis I., Hirtz C., Lehmann S., Marchi N., Janigro D., MacLeod D.B. (2022). Hypoxemia increases blood-brain barrier permeability during extreme apnea in humans. J. Cereb. Blood Flow Metab..

[56] Dohgu S., Banks W.A. (2013). Brain pericytes increase the lipopolysaccharide-enhanced transcytosis of HIV-1 free virus across the in vitro blood-brain barrier: Evidence for cytokine-mediated pericyte-endothelial cell crosstalk. Fluids Barriers CNS.

[57] Muldoon L.L., Pagel M.A., Kroll R.A., Roman-Goldstein S., Jones R.S., Neuwelt E.A. (1999). A physiological barrier distal to the anatomic blood-brain barrier in a model of transvascular delivery. AJNR Am. J. Neuroradiol..

[58] Tanaka N., Kida S., Kinoshita M., Morimoto H., Shibasaki T., Tachibana K., Yamamoto R. (2018). Evaluation of cerebrospinal fluid heparan sulfate as a biomarker of neuropathology in a murine model of mucopolysaccharidosis type II using high-sensitivity LC/MS/MS. Mol. Genet. Metab..

[59] Morimoto H., Kida S., Yoden E., Kinoshita M., Tanaka N., Yamamoto R., Koshimura Y., Takagi H., Takahashi K., Hirato T. (2021). Clearance of heparan sulfate in the brain prevents neurodegeneration and neurocognitive impairment in MPS II mice. Mol. Ther..

[60] Yamamoto R., Yoden E., Tanaka N., Kinoshita M., Imakiire A., Hirato T., Minami K. (2021). Nonclinical safety evaluation of pabinafusp alfa, an anti-human transferrin receptor antibody and iduronate-2-sulfatase fusion protein, for the treatment of neuronopathic mucopolysaccharidosis type II. Mol. Genet. Metab. Rep..

[61] Morimoto H., Imakiire A.M.H., Yamamoto R., Hirato T., Sonoda H., Minami K. (2022). Dose-dependent effects of a brain-penetrating iduronate-2-sulfatase on neurobehavioral impairments in mucopolysaccharidosis II mice. Mol. Ther..

[62] Okuyama T., Eto Y., Sakai N., Minami K., Yamamoto T., Sonoda H., Yamaoka M., Tachibana K., Hirato T., Sato Y. (2019). Iduronate-2-Sulfatase with Anti-human Transferrin Receptor Antibody for Neuropathic Mucopolysaccharidosis II: A Phase 1/2 Trial. Mol. Ther..

[63] Okuyama T., Eto Y., Sakai N., Nakamura K., Yamamoto T., Yamaoka M., Ikeda T., So S., Tanizawa K., Sonoda H. (2021). A Phase 2/3 Trial of Pabinafusp Alfa, IDS Fused with Anti-Human Transferrin Receptor Antibody, Targeting Neurodegeneration in MPS-II. Mol. Ther..

[64] Giugliani R., Martins A.M., So S., Yamamoto T., Yamaoka M., Ikeda T., Tanizawa K., Sonoda H., Schmidt M., Sato Y. (2021). Iduronate-2-sulfatase fused with anti-hTfR antibody, pabinafusp alfa, for MPS-II: A phase 2 trial in Brazil. Mol. Ther..

[65] Giugliani R., Martins A.M., Okuyama T., Eto Y., Sakai N., Nakamura K., Morimoto H., Minami K., Yamamoto T., Yamaoka M. (2021). Enzyme Replacement Therapy with Pabinafusp Alfa for Neuronopathic Mucopolysaccharidosis II: An Integrated Analysis of Preclinical and Clinical Data. Int. J. Mol. Sci..

[66] Kesik-Brodacka M. (2018). Progress in biopharmaceutical development. Biotechnol. Appl. Biochem..

[67] Luria-Perez R., Helguera G., Rodriguez J.A. (2016). Antibody-mediated targeting of the transferrin receptor in cancer cells. Bol. Med. Hosp. Infant. Mex..

[68] Leoh L.S., Daniels-Wells T.R., Martinez-Maza O., Penichet M.L. (2015). Insights into the effector functions of human IgG3 in the context of an antibody targeting transferrin receptor 1. Mol. Immunol..

[69] Liu R., Oldham R.J., Teal E., Beers S.A., Cragg M.S. (2020). Fc-Engineering for Modulated Effector Functions-Improving Antibodies for Cancer Treatment. Antibodies.

[70] Van der Horst H.J., Nijhof I.S., Mutis T., Chamuleau M.E.D. (2020). Fc-Engineered Antibodies with Enhanced Fc-Effector Function for the Treatment of B-Cell Malignancies. Cancers.

[71] Giugliani R., Giugliani L., de Oliveira Poswar F., Donis K.C., Corte A.D., Schmidt M., Boado R.J., Nestrasil I., Nguyen C., Chen S. (2018). Neurocognitive and somatic stabilization in pediatric patients with severe Mucopolysaccharidosis Type I after 52 weeks of intravenous brain-penetrating insulin receptor antibody-iduronidase fusion protein (valanafusp alpha): An open label phase 1–2 trial. Orphanet J. Rare Dis..

[72] Boado R.J., Hui E.K., Lu J.Z., Pardridge W.M. (2012). Glycemic control and chronic dosing of rhesus monkeys with a fusion protein of iduronidase and a monoclonal antibody against the human insulin receptor. Drug Metab. Dispos..

[73] Watts R., Ho C. Interim Data from DNL310 Phase 1/2 Hunter Syndrome Patient Study. https://www.denalitherapeutics.com/sites/default/fles/2021-09/iMPS%20DNL310%20Webinar%20Final.pdf.

[74] Kaffman A., White J.D., Wei L., Johnson F.K., Krystal J.H. (2019). Enhancing the Utility of Preclinical Research in Neuropsychiatry Drug Development. Methods Mol. Biol..

[75] Schulz P. (2019). Opportunities and challenges in psychopharmacology. Dialogues Clin. Neurosci..

[76] Daher A.S., Martins A.M. (2022). New hope for an old battle: Fighting Hunter disease. J. Paediatr. Child Health.

[77] Bowlby J. (1969). Attachment.

[78] Pardridge W.M. (2011). Drug transport in brain via the cerebrospinal fluid. Fluids Barriers CNS.

[79] Chang H.Y., Wu S., Chowdhury E.A., Shah D.K. (2022). Towards a translational physiologically-based pharmacokinetic (PBPK) model for receptor-mediated transcytosis of anti-transferrin receptor monoclonal antibodies in the central nervous system. J. Pharmacokinet. Pharmacodyn..

[80] Ghadessi M., Tang R., Zhou J., Liu R., Wang C., Toyoizumi K., Mei C., Zhang L., Deng C.Q., Beckman R.A. (2020). A roadmap to using historical controls in clinical trials—by Drug Information Association Adaptive Design Scientific Working Group (DIA-ADSWG). Orphanet J. Rare Dis..

[81] Shapiro E.G., Eisengart J.B. (2021). The natural history of neurocognition in MPS disorders: A review. Mol. Genet. Metab..

[82] Doessegger L., Banholzer M.L. (2015). Clinical development methodology for infusion-related reactions with monoclonal antibodies. Clin. Transl. Immunol..

[83] Kishnani P.S., Dickson P.I., Muldowney L., Lee J.J., Rosenberg A., Abichandani R., Bluestone J.A., Burton B.K., Dewey M., Freitas A. (2016). Immune response to enzyme replacement therapies in lysosomal storage diseases and the role of immune tolerance induction. Mol. Genet. Metab..

[84] Broomfield A., Jones S.A., Hughes S.M., Bigger B.W. (2016). The impact of the immune system on the safety and efficiency of enzyme replacement therapy in lysosomal storage disorders. J. Inherit. Metab. Dis..

[85] Laragh J.H., Lamport B., Sealey J., Alderman M.H. (1988). Diagnosis ex juvantibus. Individual response patterns to drugs reveal hypertension mechanisms and simplify treatment. Hypertension.

[86] Pardridge W.M. (2017). Delivery of Biologics Across the Blood-Brain Barrier with Molecular Trojan Horse Technology. Biodrugs.

[87] Parini R., Deodato F. (2020). Intravenous Enzyme Replacement Therapy in Mucopolysaccharidoses: Clinical Effectiveness and Limitations. Int. J. Mol. Sci..

[88] Burton B.K., Jego V., Mikl J., Jones S.A. (2017). Survival in idursulfase-treated and untreated patients with mucopolysaccharidosis type II: Data from the Hunter Outcome Survey (HOS). J. Inherit. Metab. Dis..

